# Development of a short form of the Cardiac Distress Inventory

**DOI:** 10.1186/s12872-023-03439-w

**Published:** 2023-08-18

**Authors:** Michael R. Le Grande, Barbara M. Murphy, Michelle C. Rogerson, Chantal F. Ski, John Amerena, Julian A. Smith, Valerie Hoover, Marlies E. Alvarenga, Rosemary O. Higgins, David R. Thompson, Alun C. Jackson

**Affiliations:** 1 Australian Centre for Heart Health, Melbourne, VIC Australia; 2 Melbourne Centre for Behaviour Change, School of Psychological Sciences, University of Melbourne, Melbourne, VIC Australia; 3 Melbourne School of Psychological Sciences, University of Melbourne, Parkville, VIC Australia; 4 Integrated Care Academy, University of Suffolk, Ipswich, UK; 5 Department of Psychiatry, University of Melbourne, Melbourne, VIC Australia; 6 Barwon Health, Geelong, VIC Australia; 7 Deakin School of Medicine, University Hospital Geelong, Geelong, VIC Australia; 8 Department of Cardiothoracic Surgery, Monash Health, Clayton, VIC Australia; 9 Department of Surgery, School of Clinical Sciences at Monash Health, Monash University, Clayton, VIC Australia; 10 Victorian Heart Institute, Clayton, VIC Australia; 11 Division of Cardiovascular Medicine, Stanford University School of Medicine, Stanford, CA USA; 12 Institute of Health and Wellbeing, Federation University, Berwick, VIC Australia; 13 Monash Health & Department of Medicine, Monash University, Clayton, VIC Australia; 14 Department of Physiotherapy, University of Melbourne, Parkville, VIC Australia; 15 School of Nursing and Midwifery, Queen’s University Belfast, Belfast, UK; 16 Centre on Behavioral Health, University of Hong Kong, Hong Kong, China

**Keywords:** Psychological distress, Cardiovascular disease, Mental health, Cardiac distress inventory, Screening

## Abstract

**Background:**

Cardiac distress may be viewed as a persistent negative emotional state that spans multiple psychosocial domains and challenges a patient’s capacity to cope with living with their heart condition. The *Cardiac Distress Inventory* (CDI) is a disease-specific clinical assessment tool that captures the complexity of this distress. In busy settings such as primary care, cardiac rehabilitation, and counselling services, however, there is a need to administer briefer tools to aid in identification and screening. The aim of the present study was to develop a short, valid screening version of the CDI.

**Methods:**

A total of 405 participants reporting an acute coronary event in the previous 12 months was recruited from three hospitals, through social media and by direct enrolment on the study website. Participants completed an online survey which included the full version of the CDI and general distress measures including the Kessler K6, Patient Health Questionnaire-4, and Emotion Thermometers. Relationship of the CDI with these instruments, Rasch analysis model fit and clinical expertise were all used to select items for the short form (CDI-SF). Construct validity and receiver operating characteristics in relation to the Kessler K6 were examined.

**Results:**

The final 12 item CDI-SF exhibited excellent internal consistency indicative of unidimensionality and good convergent and discriminant validity in comparison to clinical status measures, all indicative of good construct validity. Using the K6 validated cutoff of ≥ 18 as the reference variable, the CDI-SF had a very high Area Under the Curve (AUC) (AUC = 0.913 (95% CI: 0.88 to 0.94). A CDI-SF score of ≥ 13 was found to indicate general cardiac distress which may warrant further investigation using the original CDI.

**Conclusion:**

The psychometric findings detailed here indicate that the CDI-SF provides a brief psychometrically sound screening measure indicative of general cardiac distress, that can be used in both clinical and research settings.

**Supplementary Information:**

The online version contains supplementary material available at 10.1186/s12872-023-03439-w.

## Background

The many challenging emotions, changes and experiences that follow an acute cardiac event have been conceptualised as ‘cardiac distress’. Cardiac distress is defined as ‘’*a persistent negative emotional state rather than a transient state; involving multiple psychosocial domains; that challenges a patient’s capacity to cope with living with their heart condition, the treatment of the condition, and the resultant changes to daily living; and challenges the person’s sense of self and future orientation’’* [1] In recognition of the fact that cardiac distress spans multiple psychosocial domains and that existing psychosocial measures were deemed not to provide a comprehensive or detailed assessment of cardiac distress, the 55 item Cardiac Distress Inventory long form (CDI) was developed [2, 3]. Instrument development followed best practice instrument design principles based on a strong conceptual [1], clinical [4] and empirical base [5, 6].

The 8 subscale CDI has broad utility for use in both research and clinical practice. In research, the full CDI can be used to provide an overall measure of distress, while individual subscales can be used to provide assessment of specific areas of distress. In clinical practice, the full inventory can be administered to assist practitioners to identify specific areas of concern for their patients or, where indicated, individual subscales could be used to assess or monitor specific areas of distress. The CDI is already proving [7] to have great utility in assisting practitioners to target and tailor therapeutic interventions to relevant areas of need for individual patients. The 55-item CDI is regarded as a clinical *assessment* tool.

There is also a need for a briefer tool to *screen* for cardiac distress. The use of brief screening tools is proposed as a means of improving the identification and management of mental health problems and is indicated in settings where time limitations point to the need for briefer instruments [8]. A disease-specific screening tool for cardiac distress could be used in settings such as primary care, cardiac rehabilitation (CR), and counselling services. Therefore, the aim of this study was to develop a screening version of the 55 item CDI by extracting items representative of all 8 sub-scales, to be known as the CDI-SF.

## Methods

### Participants

The item selection process, participant recruitment and psychometric testing of the *Cardiac Distress Inventory* (CDI) are described in full elsewhere [2]. Development of the CDI-SF involved a secondary data analysis based on the data collected from the same sample that was used to develop the CDI [2]. Four hundred and five people were recruited for the study during the COVID-19 pandemic (between October 2020 and November 2021): 231 from hospitals based in Australia and the United States (57.0%), and 174 (43.0%) through convenience sampling following social media promotion of the study. Age of participants ranged from 22 to 90 years (median 62 years) with 53% male and 47% female participants. Participants were eligible to take part in the study provided they had an acute coronary event namely acute coronary syndrome (ACS), acute myocardial infarction (AMI) or coronary artery bypass graft surgery (CABGS) in the previous 6 months. Patients who did not have adequate English language proficiency to read and understand the Patient Information and Consent Form and questionnaire were excluded.

.

### Measures

#### Cardiac distress inventory

The CDI is a 55-item measure comprising eight subscales, with the number of items varying across the subscales. The subscales assess Fear and uncertainty (8 items), Disconnection and hopelessness (8 items), Changes to roles and relationships (11 items), Overwhelm and depletion (7 items), Cognitive challenges (4 items), Physical challenges (8 items), Health system challenges (5 items), and Death concerns (4 items) [2]. A two-step response scale is used: first, items are endorsed as present or absent; second, for items endorsed as present, distress severity is assessed on a 4-point scale ranging from 0 ‘no distress’ to 4 ‘severe distress’. Validation of the CDI against the *Kessler Psychological Distress Scale-6* (K6) [2] supported its criterion validity. Evidence of concurrent validity of the total CDI score was provided by the high correlation obtained with *Emotion Thermometer* ratings [9]. The CDI total score provided good overall discriminative accuracy relative to the established cutoff scores of the *Emotion Thermometers.*

#### Clinical status

*Kessler K6*: Self-reported psychological distress was assessed using the Kessler Psychological Distress Scale (K6) [10]. The K6 comprises six items that assess general psychological distress in the past 30 days. Items (e.g. ‘How often did you feel hopeless?’) are scored on a 5-point Likert-type scale ranging from 1 ‘none of the time’ to 5 ‘all of the time’ are summed to provide a total score for psychological distress (Australian scoring range: 6–30). A higher score indicates poorer mental health status. The cutoff score for detecting the possibility of severe psychological distress is ≧ 19 [11]. The K6 has good psychometric properties [10], including good internal reliability in the study sample (Chronbach Alpha = 0.88).

*Patient Health Questionnaire-4* (PHQ-4): The PHQ-4 [12] is a validated brief screener (4-items) for anxiety and depression, which combines the Patient Health Questionnaire-2 (PHQ-2) and the Generalized Anxiety Disorder-2 (GAD-2) [12]. Total scores range from 0 to 12, with 0 indicating ‘no distress’ and 12 indicating ‘severe distress’. The PHQ-4 has good reliability with pre-operative surgical patients [13] and has good prognostic value with CVD patients [14].

##### Emotion thermometers

Single-item Emotion Thermometers were used to assess distress, anxiety, depression and anger. Each consists of a “thermometer” with numerals displayed vertically from 0 to 10. For example, on the Distress Thermometer, patients rate their distress “over the last week” anywhere from 0 to 10, with 0 indicating “no distress” and 10 indicating “high distress”. A total score from all four emotion thermometers indicates overall emotional problems. The thermometers, based on the NCCN cancer distress thermometer (DT) [9], have been shown to be a clinically sensitive measure of distress in patients with mixed cardiovascular conditions [15].

##### COVID-19 concern

Using the same response format as the Emotion Thermometers, participants were asked to rate their concern or anxiety about the coronavirus disease (COVID-19) situation over the past week. This question was asked to assist with ascertaining the degree to which participant-reported distress may have been attributable to the experience of living through the COVID-19 pandemic.

### Data Analysis

#### CDI Short-Form development

After examination of the long form CDI [2] factor structure and loadings it was decided *apriori* that at least 12 items from all 8 CDI sub-scales would be required for inclusion in the short-form scale to fully represent the multifactorial nature of cardiac distress. Inclusion of items from all dimensions of the original instrument is a standard technique in short-form development [16–18]. Rather than simply allocating two or more items per dimension to the short-form, we weighted the short-form so that the more influential factors would have more items, consistent with the factor structure of the long form CDI. Thus, to retain the theoretical structure of the CDI, two items per CDI subscale were selected from the top four loading factors obtained through the initial CDI exploratory factor analysis (subscales 1 thru 4) where most variance was obtained, with one item per subscale chosen for the less influential factors (subscales 5 thru 8).

#### Relationship to clinical status

It was decided *apriori* that the relationship of items to clinical status was the most important consideration for inclusion in the short form, followed by psychometric characteristics and clinical judgement. We first assessed the Spearman’s correlation for each item of the CDI with the clinical status measures: K6 total score and the Distress Thermometer from the Emotion Thermometer suite. We then took the top two correlating items from each of the 8 subscales. If the item correlated the highest on both measures the item was included without further examination. If the item only correlated highest on one of the ‘gold standards’ or if there were borderline items, we examined other psychometric characteristics based on Rasch modelling.

### Rasch Psychometrics

Rasch fit indices as calculated within each scale for the long form CDI were used to evaluate item properties and further rank suitability of items for inclusion in the short form. Good fit indices indicate that an item fits to the Rasch model’s expectations based on item difficulty (endorsement) and subjects’ ability level. Fit indices rely on the mean square of the standardized residuals for items, which are not sample-size dependent. The expected value for fit indices for rating scales is 1, but recommended values should not exceed 1.4 and not be lower than 0.7 [19]. Values above 1.4 suggest that there is unexplained variance (i.e., underfit), while values below 0.7 mean that there is redundancy among the items (i.e., overfit). Items showing outfit values over 1.4 were not considered for the short form, with items with fit indices closer to 1 considered to be more appropriate for inclusion. Differential Item Functioning (DIF) was analysed to test the invariance of each item across different groups of subjects (e.g. females and males). An item exhibiting problematic DIF is answered differently by subjects with the same ability level (in this case we matched on CDI long form sub-scale total score). Adverse DIF may occur, for example, because of different understandings of a word or phrase used in the item and differences in item response may reflect artifactual elements [20] rather than the intended measurement of cardiac distress. Items with large effect sizes (difference of at least 0.5 logits [21]) for DIF were considered unsuitable for short form selection.

### Clinical Oversight

Our approach also involved exercising theoretical and clinical judgement. We referred borderline cases to a Clinical Expert Committee comprising experienced clinicians employed at the Australian Centre for Heart Health Cardiac Counselling Clinic. For example, in one instance where there was very little to separate the candidate items in terms of psychometrics and relationship to clinical status, we considered whether the items in question were suitable for the entire cardiac population or to some segments only.

### Construct validity

Given significant non-normality of the data, we correlated the CDI-SF with the full CDI using Spearman’s correlation coefficients. We expected convergent validity (strong correlations of at least 0.7) of the CDI-SF total score with the K6, PHQ2, GAD2 (PHQ4) and the Distress, Anxiety and Depression Thermometers. We expected to demonstrate discriminant validity by obtaining lower correlations on scales that may not necessarily correlate well with cardiac distress such as COVID-19 Concern and the Anger Thermometer.

### Screening utility

We determined cutoff scores to identify “cases” with moderate/severe symptoms for further assessment using the K6 cutoff score of ≥ 18 as the gold standard [11]. These dichotomized scores were used to determine the optimal cutoff score for CDI-SF by calculating the Receiver Operating Characteristic (ROC) curve, of which an Area Under the Curve (AUC) larger than 0.7 indicates acceptable diagnostic ability [22]. We also assessed performance of the various cutoff levels by calculating sensitivity, specificity, positive and negative predictive value (PPV/NPV), positive and negative likelihood ratio values (LR +/LR −). The LR is the likelihood that a given test result would be expected in a patient with the target disorder compared to the likelihood that that same result would be expected in a patient without the target disorder. Instruments with high LR + values and very low LR − have greater discriminating ability [23]. We computed the 95% confidence interval for optimal cutoff scores using bootstrapping techniques with the ‘cutpt’ Stata addon. We chose the method that compares every possible cutoff point and selects the point closest-to-(0,1) corner or perfection in the ROC plane thus minimising misclassification [24]. We also compared this method with a more recent approach that defines the optimal cutoff value as the value whose sensitivity and specificity are the closest to the value of the area under the ROC curve [25]. Finally, we compared the two derived groupings (below and above the clinical cutpoint) on age, sex, psychosocial outcomes (K6, PHQ2, GAD2, Emotion Thermometers) and Covid Concern.

We presented sociodemographic and clinical characteristics of participants (Table 1) as frequency and percentage. We used Pearson Chi Square to examine categorical associations and One Way Analysis of Variance for comparison of continuous measures. Alpha was set at 0.05 and for Table 2 was adjusted for false discovery rate using the Benjamin-Hochberg calculation [26].Analyses were conducted using Stata 16.1 (Stata Corp, College Station, TX, USA).

**Table 1:** Sociodemographic and clinical characteristics of participants

Characteristic	N = 405
Female	188 (47%)
Age Group (years)	
< 50	48 (12%)
50–59	109 (27%)
60–69	134 (33%)
≥ 70	112 (28%)
Education	
Primary or Secondary	130 (32%)
Trade qualification	103 (26%)
University diploma/degree/post-graduate	170 (42%)
Not in the paid workforce (e.g. home duties, retired)	177 (44%)
Financial Strain Experienced	
None or slight	216 (55%)
Moderate	126 (32%)
Considerable/Extreme	48 (12%)
Lives Alone	74 (18%)
Has Close Confidante	334 (85%)
Married or living with partner	288 (71%)
Heart Condition	
AMI	162 (40%)
Heart Failure	30 (7.4%)
Atrial Fibrillation	61 (15.1%)
SCAD	39 (9.6%)
Treatment for Heart Condition*	
CABGS	118 (29%)
PCI	299 (74%)
ICD	8 (2.0%)
*Comorbidities*	
Obesity	61 (15%)
Diabetes	86 (21%)
Sleep Disorder	53 (13%)
Cancer	22 (5.4%)
History of Anxiety	101 (25%)
History of Depression	117 (29%)
Time Since Cardiac Event	
Less than 1 month	45 (11%)
1 to 3 months	239 (59%)
4 to 12 months	67 (16.5%)
More than 1 year	50 (12.3%)
Attended Cardiac Rehabilitation	179 (46%)

Note: Not all categories add to 405 due to missing data;* not mutually exclusive; Rasch Analysis was conducted on sub-sample with no missing data on the CDS Inventory (n-385, 95% of total sample)

**Table 2:** Age, sex and clinical status as a function of CDI-SF clinical categories

	CDI-SF clinical cutoff			
	Non-distressed	Distressed		
	Mean (SD)	Mean (SD)		Test Statistic, df, p value
N	288	117		
Age	63.1 (11.9)	59.8 (10.6)		F_1,401_=8.51, 0.009^1^
Sex				Χ21 = 17.58 < 0.001^2^
Male	173.0 (60.1%)	43.0 (37.1%)		
Female	115.0 (39.9%)	73.0 (62.9%)		
Kessler K6	10.2 (3.6)	17.6 (4.6)		F_1,391_=255.54,< 0.001^1^
PHQ2	1.0 (1.3)	3.2 (1.7)		F_1,386_=193.08,< 0.001^1^
GAD2	1.1 (1.3)	3.2 (1.7)		F_1,386_=165.44,< 0.001^1^
Therm - Distress	1.7 (2.2)	5.3 (2.5)		F_1,386_=187.05,< 0.001^1^
Therm -Anxiety	2.4 (2.6)	6.0 (2.4)		F_1,386_=153.04,< 0.001^1^
Therm -Depression	1.6 (2.4)	5.3 (2.8)		F_1,386_=182.06,< 0.001^1^
Therm - Anger	1.7 (2.3)	4.4 (2.8)		F_1,386_=111.21,< 0.001^1^
COVID concern	2.9 (2.9)	4.8 (3.2)		F_1,365_=31.29, < 0.001^1^

1 Linear Model ANOVA 2.Pearson’s Chi-squared test

All comparisons were statistically significant following the Benjamini-Hochberg adjustment calculation

## Results

Sociodemographic and clinical characteristics of participants are presented in Table 1. Briefly, characteristics generally matched those of Australians with cardiovascular disease [27], with a greater representation of males and those aged 60 and over, with close to half not being in the paid workforce. Over a third of participants had experienced an AMI which was often treated by either CABGS (21% of AMI patients) or more commonly, percutaneous coronary intervention (PCI) (41% of AMI patients). Over two-thirds of participants had experienced their cardiac event within the preceding three months. Over a third of participants reported either a history of anxiety or depression. Close to half of participants reported having attended cardiac rehabilitation.

### Selection of items for the CDI Short Form

The relationship between the 55 CDI items and clinical status, and Rasch fit statistics, are presented in Table 3. Within each CDI subscale we highlighted the two highest item-clinical status Spearman correlation coefficients with the items chosen for the CDI-SF denoted with an asterisk. With some subscales, item selection was clearly indicated with consistently high correlations with all or most clinical status measures and acceptable Rasch psychometric characteristics. For example, in the Disconnection and Hopelessness subscale, the items “feeling lonely” and “withdrawing from people” highly correlated on all four of the clinical status measures and had good Rasch fit. In other subscales the item selection decision was less clear cut. For example, in the Fear and Uncertainty subscale the item “Thinking that I am not the person that I used to be” was a strong candidate for short form selection with little to separate that item in terms of clinical status correlations and Rasch fit from “Thinking I will never be the same again”. In this specific case we chose the latter item based on clinical experience in consultation with our Clinical Expert Committee. In some instances, non-selection in terms of relationship with clinical status was vindicated with reference to Rasch fit. For example, in the Cognitive Challenges subscale, “Having difficulty making decisions” had relatively strong correlations with clinical status, but not the highest, and had relatively poor Rasch infit, outfit and larger DIF indicating unsuitability for selection in the CDI-SF. The final 12-item CDI-SF is presented in Additional file 1. With the current sample of cardiac patients, the CDI-SF had a mean score of 9.2 (sd = 7.9) (range 0 to 36) and mean standardized score (out of 100) of 25.7 (sd = 22.0). Internal consistency was good with a Chronbach Alpha of 0.90.

**Table 3:** CDI item relationship with clinical status and Rasch psychometric characteristics

	Correlation with Clinical Status				Rasch Psychometrics		
**CDI Sub-Scale**	Kessler K6	Distress Thermometer	PHQ2	GAD2	Infit	Outfit	DIF (Sex) Effect size
**Fear and uncertainty**							
Thinking that I am not the person that I used to be	**0.585**	0.491	**0.530**	**0.556**	0.86	0.81	− 0.11
Thinking my condition might get worse	0.432	0.454	0.394	0.477	1.02	1.09	0.14
Being in places and situations that remind me of my heart event	0.368	0.329	0.314	0.321	1.45	1.08	0.11
Avoiding activities that make my heart beat faster	0.436	0.400	0.351	0.401	1.09	1.14	0.02
Dwelling on my heart condition	0.486	0.481	.**466**	0.516	1.09	0.92	− 0.17
Being unable to plan for the future	0.564	0.485	0.443	0.477	1.11	0.98	− 0.08
*Thinking I will never be the same again	0.575	**0.522**	0.465	**0.556**	0.79	0.84	− 0.05
*Not knowing what the future holds for me	**0.590**	**0.539**	0.455	**0.557**	0.86	0.84	0.13
**Disconnection and hopelessness**							
Thinking my friends or family don’t understand how difficult it is living with heart disease	0.433	0.461	0.403	0.422	0.97	0.94	0.18
Being disconnected from people in my community	0.420	0.349	0.475	0.398	0.78	0.65	− 0.01
Being isolated from friends and family	0.376	0.339	0.422	0.366	0.98	0.68	− 0.08
Believing that others don’t have the same confidence in me as they did before my heart problem	0.377	0.321	0.286	0.348	1.34	1.32	− 0.15
Not being supported by my friends and family in my efforts to manage my heart condition	0.351	0.333	0.326	0.327	0.78	0.63	0.05
Being unable to accept help from others	0.428	0.354	0.377	0.403	1.46	1.56	0.09
*Feeling lonely	**0.544**	**0.464**	**0.588**	**0.485**	0.89	0.90	0.01
*Withdrawing from people	**0.581**	**0.518**	**0.555**	**0.572**	0.84	0.83	− 0.11
**Changes to roles and relationships**							
Not being able to return to work or continue working	0.343	0.320	0.344	0.325	1.24	1.17	− 0.03
Not being able to go too far from home	0.457	0.370	0.383	0.364	1.03	0.98	0.06
Being unable to take care of family responsibilities	0.467	0.418	0.363	0.402	0.85	0.82	0.08
Being concerned about my capacity for sexual activity	0.321	0.301	0.326	0.269	1.30	1.48	− 0.37
Being unavailable to my family and friends	0.432	0.386	0.390	0.396	0.91	0.74	0.12
Being too dependent on others	0.390	0.293	0.314	0.316	0.90	0.76	0.06
Becoming a burden to my family	0.490	0.432	0.440	0.462	0.94	0.88	0.13
Thinking that my family is being overprotective of me	0.255	0.186	0.163	0.171	1.23	1.54	− 0.07
Thinking that my heart condition controls my life	0.506	**0.460**	**0.473**	**0.456**	0.84	0.82	0.06
*Having changes in my usual roles	**0.525**	0.448	0.430	0.405	0.72	0.76	0.12
*Lacking purpose or meaning in life	**0.568**	**0.477**	**0.570**	**0.465**	1.08	0.99	− 0.18
**Overwhelm and depletion**							
Being tearful more easily than before	0.463	0.437	0.450	0.446	1.14	0.65	0.03
Avoiding situations and activities	0.534	0.500	0.502	0.546	0.97	1.01	0.05
Being irritated by little things	0.517	0.449	0.495	0.496	0.86	0.86	− 0.23
Not being able to sustain the lifestyle changes I need to make	0.487	0.440	0.429	0.413	1.22	1.15	0.05
Lacking energy	0.617	0.512	0.505	0.497	1.08	1.17	0.03
*Being unable to deal with stress	0.598	**0.525**	**0.553**	**0.603**	1.09	0.93	0.02
*Being emotionally exhausted	**0.683**	**0.629**	**0.613**	**0.619**	0.76	0.69	0.09
**Cognitive challenges**							
Having difficulty making decisions	**0.485**	**0.414**	**0.448**	**0.421**	1.41	1.29	0.11
Having difficulty remembering things	0.417	0.360	0.397	0.390	0.94	0.91	0.08
Forgetting things more than before	0.426	0.370	0.417	0.382	0.73	0.72	− 0.07
*Having difficulty concentrating	**0.541**	**0.475**	**0.502**	**0.507**	0.99	1.03	0.02
**Physical challenges**							
Being woken up at night by my racing heart	0.296	0.257	0.167	0.234	1.23	1.01	0.04
Having more pain than I can deal with	0.229	0.283	0.191	0.208	1.45	1.05	0.06
Having bad dreams or nightmares	0.409	0.315	0.370	0.336	1.42	1.17	− 0.09
Being overly aware of my heart in my chest	**0.465**	0.465	0.353	**0.486**	0.89	0.90	0.25
Having chest discomfort	0.399	0.353	0.357	0.350	0.79	0.76	0.10
Being short of breath	0.235	0.151	0.203	0.183	1.15	1.27	− 0.28
Not sleeping well	0.461	**0.448**	**0.440**	0.432	0.98	0.98	− 0.12
*Being physically restricted	**0.544**	**0.506**	**0.503**	**0.479**	0.74	0.74	0.05
**Health system challenges**							
Not having access to the health care I need	0.273	0.287	**0.268**	0.223	1.08	0.97	− 0.11
Having difficulty getting to appointments that I need to attend	0.283	0.236	0.198	0.180	1.35	1.40	− 0.04
Not being able to get as much information as I want about my heart condition	**0.299**	**0.293**	0.263	**0.303**	0.96	0.91	0.07
Not having my concerns taken seriously by my health practitioner	0.275	0.244	0.211	0.239	1.01	0.90	0.12
*Not getting clear directions from my health practitioner on how to manage my heart condition	**0.346**	**0.339**	**0.269**	**0.282**	0.73	0.68	− 0.04
**Death concern**							
Not knowing what will happen to other people if I die	**0.543**	0.387	0.306	0.425	1.03	0.93	− 0.21
Not knowing how my family will cope if something should happen to me	0.503	0.371	0.294	0.387	1.07	1.03	0.05
Being afraid of dying	0.529	**0.408**	**0.355**	**0.464**	1.00	0.95	0.11
*Thinking about dying	**0.607**	**0.437**	**0.422**	**0.511**	0.90	0.86	0.05

Spearman correlations; Top 2 correlations within each sub-scale highlighted in bold; * items selected for short-form

### Construct validity

The Spearman correlation coefficients between the CDI-SF, the CDI and other clinical status measures are presented in Table 4. There was a very high positive correlation between the short and long forms of the CDI (*r* = 0.96, p < 0.001). Evidence of convergent validity was confirmed with strong positive correlations of the CDI-SF with the three clinical status measures, highest with the K6. The CDI-SF correlated strongly with the distress, anxiety, and depression thermometers, and only moderately with the Anger and COVID-19 thermometers, supporting its discriminant validity.

**Table 4:** Spearman correlation coefficients and significance between key variables (N = 393)

	CDI-SF	CDI	K6	PHQ2	GAD2	ET-Distress	ET-Anxiety	ET-Depression	ET-Anger	COVID
**CDI-SF**	—									
**CDI**	0.964	—								
	< .001	—								
**K6**	0.808	0.801	—							
	< .001	< .001	—							
**PHQ2**	0.743	0.729	0.773	—						
	< .001	< .001	< .001	—						
**GAD2**	0.763	0.757	0.78	0.717	—					
	< .001	< .001	< .001	< .001	—					
**ET-Distress**	0.736	0.726	0.736	0.663	0.716	—				
	< .001	< .001	< .001	< .001	< .001	—				
**ET-Anxiety**	0.736	0.721	0.725	0.669	0.758	0.808	—			
	< .001	< .001	< .001	< .001	< .001	< .001	—			
**ET-Depression**	0.699	0.68	0.726	0.754	0.664	0.698	0.721	—		
	< .001	< .001	< .001	< .001	< .001	< .001	< .001	—		
**ET-Anger**	0.582	0.566	0.582	0.533	0.534	0.597	0.594	0.605	—	
	< .001	< .001	< .001	< .001	< .001	< .001	< .001	< .001	—	
	0.407	0.437	0.388	0.343	0.343	0.427	0.378	0.416	0.296	—
**COVID**	< .001	< .001	< .001	< .001	< .001	< .001	< .001	< .001	< .001	—

CDI-SF = Cardiac Distress Inventory Short Form; CDI = Cardiac Distress Inventory (55 item version); K6 = Kesler K6; ET = Emotion Thermometer; COVID = Covid-19 concern; Spearman r coefficient and *p* value shown

### Screening utility and recommended clinical cutoff of the CDI-SF

**Fig. 1:**
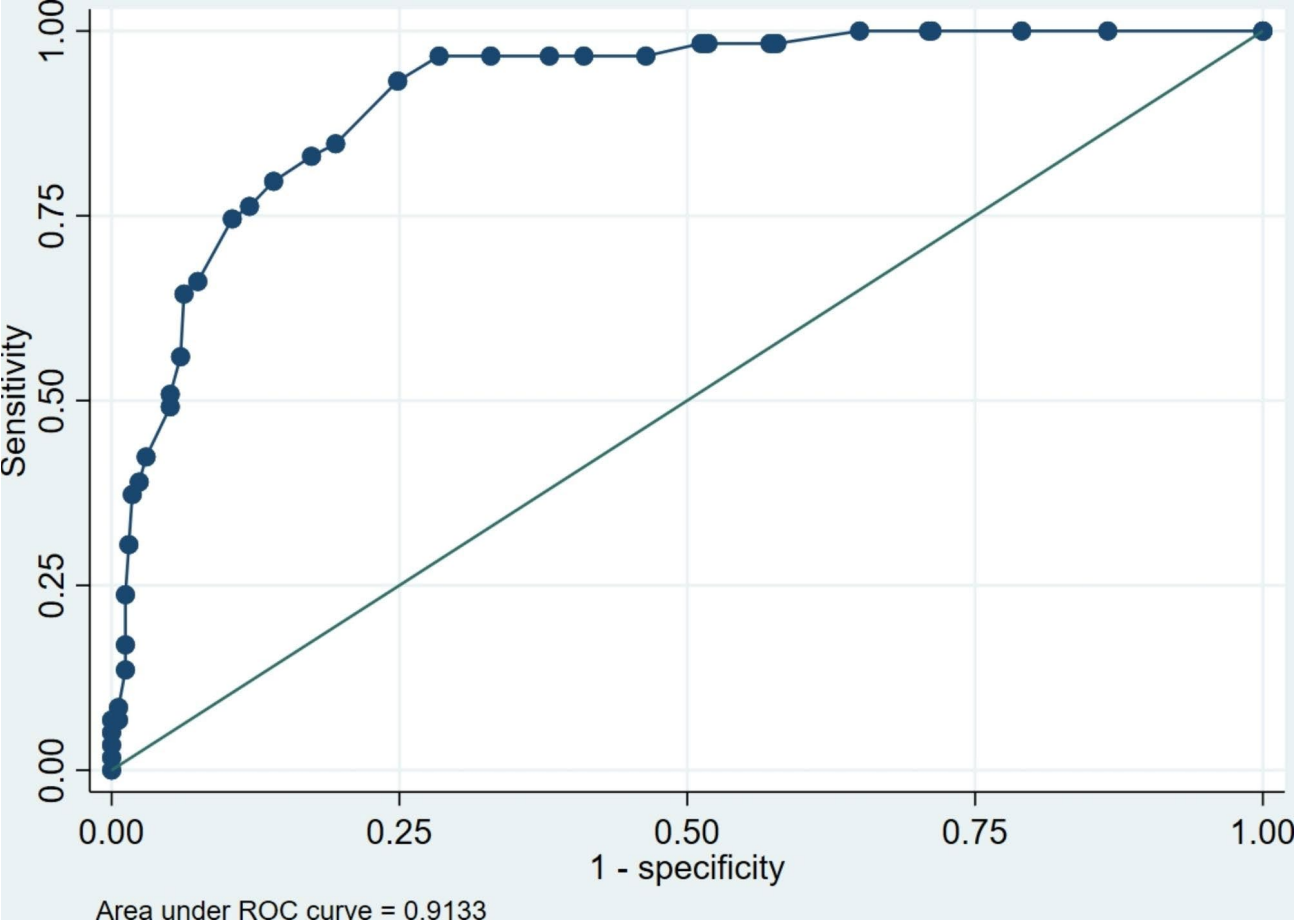
Receiver operating characteristics curve for CDI-SF scores using the Kessler K6 as the reference variable (clinical cutoff of ≥ 18 indicated probable serious mental illness)

**Fig. 2:**
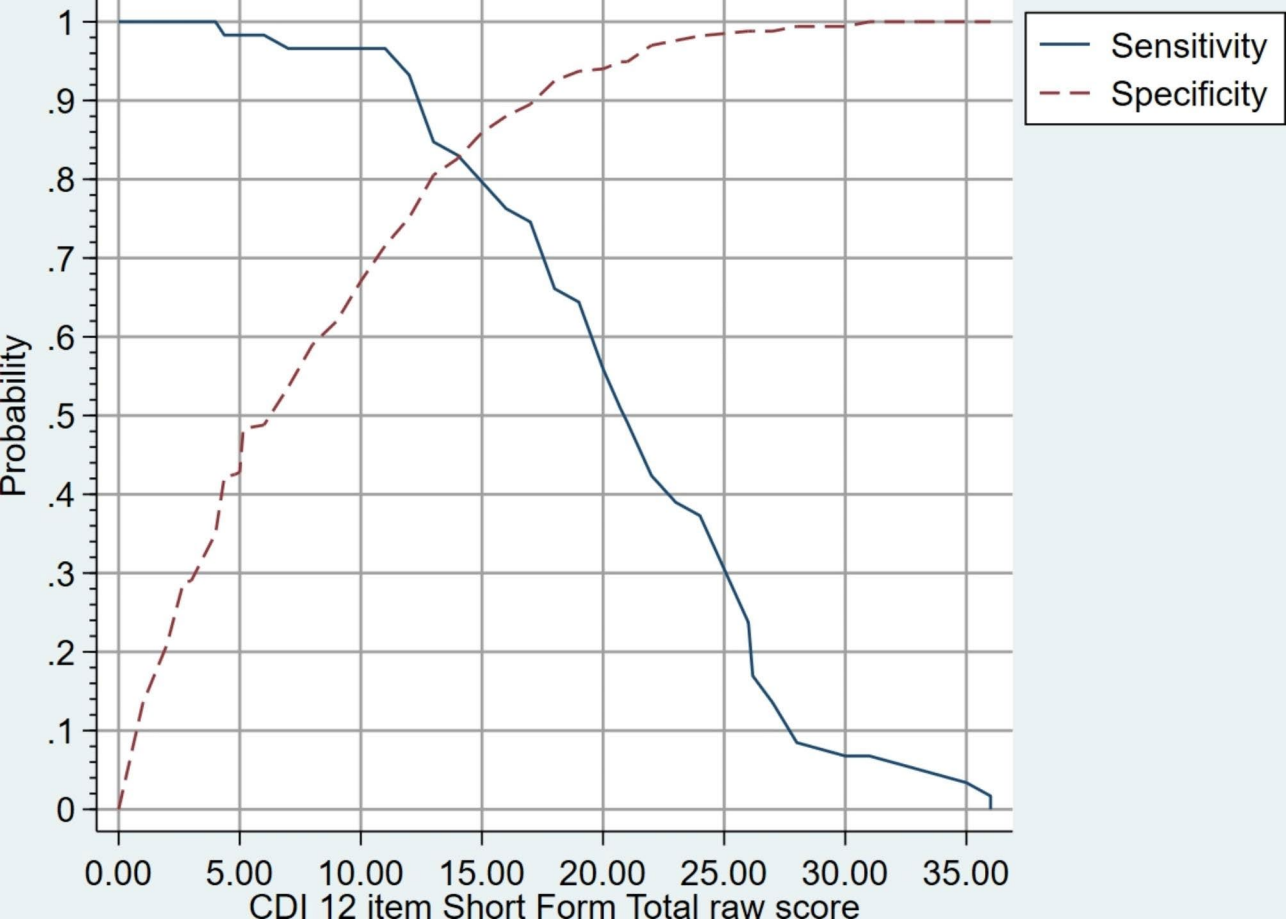
Sensitivity and specificity of the CDI-SF in prediction of probable serious mental illness as referenced by the Kessler K6 (clinical cutoff of ≥ 18 indicated probable serious mental illness)

The CDI-SF performed well in diagnostic predictability using the K6 validated cutoff of ≥ 18 as the reference variable (Area Under Curve: AUC = 0.913 (95% CI: 0.88 to 0.94)) (See Fig. 1). The optimal cutoff that minimises misclassification was a CDI-SF score of ≥ 13 (see Fig. 2). The cutoff of ≥ 13 was also selected using the method that checks the sensitivity and specificity that is closest to the value of the area under the ROC curve [25]. Detailed sensitivity, specificity, and likelihood ratio values are presented in Additional file 2. At the recommended CDI-SF cutoff score of ≥ 13 there is both acceptable sensitivity (84.8%) and specificity (80.5%) with a LR + of 4.8 and LR- of 0.2. The mean scores of clinical status measures by the recommended CDI-SF clinical cutoff are presented in Table 2. Those respondents who were deemed to be distressed were significantly more likely to be female and score higher on all the clinical status measures.

## Discussion

In this paper we report the development and validation of the CDI-SF, a 12-item screening measure of cardiac distress. Our approach throughout this process was to utilise best practice methods from both classical test theory [28] and Rasch modelling [29], with an overarching emphasis on clinical utility and practicality to drive development. The final 12-item screener comprises items to address each of the 8 subscales of the comprehensive 55-item CDI; two items each for the four primary sub-scales (Fear and uncertainty, Disconnection and hopelessness, Changes to roles and relationships, Overwhelm and depletion). and a single item each for the other four (Cognitive challenges, Physical challenges, Health system challenges Death concern).

The 12-item CDI-SF demonstrates good psychometric properties in this study. The preliminary evidence obtained here for both convergent and discriminant validity with other brief clinical status measures gives us confidence that this short form is a valid screener for cardiac distress. Our instrument at the recommended cutoff of ≥ 13 exhibited high LR + and low LR − values indicative of excellent discriminating ability [23]. In this cross-sectional study we found that the new instrument has excellent internal consistency. However, test-retest reliability, sensitivity to intervention and further aspects of validity will need to be determined and replicated in future longitudinal studies.

The very strong correlation observed between the CDI and the CDI-SF in this study indicates that the new brief measure may be a good unidimensional indicator of overall cardiac distress. We believe the original 55-item CDI [30] has immense potential for use in research and clinical settings when there is time to utilise all or some of the 8 subscales and when the objective is to evaluate cardiac distress in a more nuanced manner. In clinical settings where time and staff resources may be limited, such as CR programs, a brief instrument such as the CDI-SF may be more appropriate. In such settings, the CDI-SF may be a useful screening tool that may identify patients in need of further psychological and emotional support. The CDI-SF is also likely to be useful in research settings when a brief measure of general cardiac distress is required.

Significantly higher scores on the CDI-SF were obtained for younger patient and for females. This finding is in keeping with several studies finding higher anxiety, depression and distress in these population groups [5, 31]. Further research is required to unravel the complexity of cardiac distress experienced, as confounding factors such as diagnostic category, treatment method, and stage of illness/rehabilitation should also be considered [5, 6].

This study has several limitations. First, we developed the CDI-SF clinical cutoff score using the K6, whereas a clinical interview may represent a superior “gold standard” for comparison. However, practical considerations limited our ability to administer clinical interviews, with the K6, which has been established as a valid and reliable instrument to measure general distress, providing a more practical alternative. Further studies may elucidate the value of the disease-specific CDI-SF developed here, over and above more generic brief instruments of distress such as the K6. Regardless of what these studies may find, our main intention was to develop a short practical disease-specific screener, that could be followed up with the more nuanced CDI if necessary. Second, the CDI-SF was validated using the same patient sample on which the long form CDI was developed. This approach was taken for pragmatic reasons. Third, for the convenience sample there was no independent verification of respondents’ self-reported cardiac conditions. Importantly though, this group made up only two fifths of the study sample, with the majority being recruited as a consecutive series of hospital admissions for cardiac conditions. Nonetheless, we cannot guarantee that our sample represents the total population of patients with cardiac problems. Despite these limitations, the CDI-SF is likely to be a useful and practical screener for cardiac distress in a range of settings and diagnostic types and we welcome further research using this tool.

## Conclusion

The value of a screening tool is dependent on how well it is accepted and incorporated into clinical practice. Given competing demands and scarcity of resources and time, we believe the CDI-SF offers a brief, practical and valid indication of cardiac distress. We look forward to clinicians and researchers using this tool, adapting, and translating where necessary, to use in a variety of settings such as primary care, cardiac rehabilitation, and counselling services throughout the world. The CDI-SF also has potential applicability in large research studies where brief valid instruments are required.

### Electronic supplementary material

Below is the link to the electronic supplementary material.

**Additional file 1**: The final CDI-SF instrument

**Additional file 2**: Sensitivity and specificity for CDI-SF total scores in prediction of Kessler K6 cutoff for probable serious mental illness (K6 Australian scoring cutoff ≥18)

## Data Availability

The data sets generated during the original study will not be publicly available until December 2023 as the authors are continuing analysis of the data but will be available on the corresponding author’s organizational website (www.australianhearthealth.org.au) after that time. Data queries can be directed to the corresponding author.
