# Supplementary material for: Development of a short form of the Cardiac Distress Inventory
Source: BMC Cardiovasc Disord. 2023 Aug 18;23:408. doi: 10.1186/s12872-023-03439-w (PMC10439557; doi:10.1186/s12872-023-03439-w)
Supplement: Supplementary file 1 — Additional file 1: The final CDI-SF instrument [file 12872_2023_3439_MOESM1_ESM.docx]

**Additional file 1:** The final CDI-SF instrument


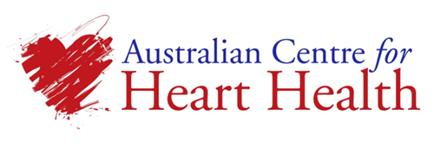


**Cardiac Distress Inventory – Short Form (CDI-SF)©**

Please indicate whether or not you have experienced each issue during the past four weeks by circling “Y” for yes or “N” for no. For each item that you have circled “Y”, indicate how much distress this issue has caused you **during the past four weeks** by circling, on a scale of 0 to 3, where “0” is no distress and “3” is severe distress.

|  | | | **If yes, indicate how much distress this has caused for you** | | | |
| --- | --- | --- | --- | --- | --- | --- |
| **Issue** | **Yes** | **No** | **No distress** | **Slight distress** | **Moderate distress** | **Severe distress** |
| 1. Thinking I will never be the same again | Y | N | 0 | 1 | 2 | 3 |
| 1. Not knowing what the future holds for me | Y | N | 0 | 1 | 2 | 3 |
| 1. Feeling lonely | Y | N | 0 | 1 | 2 | 3 |
| 1. Withdrawing from people | Y | N | 0 | 1 | 2 | 3 |
| 1. Having changes in my usual roles | Y | N | 0 | 1 | 2 | 3 |
| 1. Lacking purpose or meaning in life | Y | N | 0 | 1 | 2 | 3 |
| 1. Being unable to deal with stress | Y | N | 0 | 1 | 2 | 3 |
| 1. Being emotionally exhausted | Y | N | 0 | 1 | 2 | 3 |
| 1. Having difficulty concentrating | Y | N | 0 | 1 | 2 | 3 |
| 1. Being physically restricted | Y | N | 0 | 1 | 2 | 3 |
| 1. Not getting clear directions from my health practitioner on how to manage my heart condition | Y | N | 0 | 1 | 2 | 3 |
| 1. Thinking about dying | Y | N | 0 | 1 | 2 | 3 |

© Australian Centre for Heart Health

The scores on the CDI-SF range from 0-36. For a dichotomous measure, >13 should be seen as an indicator of distress, and an indicator that a patient should now complete the 55-item CDI long form, preferably as part of a clinical interview.
