# Supplementary material for: Development of a short form of the Cardiac Distress Inventory
Source: BMC Cardiovasc Disord. 2023 Aug 18;23:408. doi: 10.1186/s12872-023-03439-w (PMC10439557; doi:10.1186/s12872-023-03439-w)
Supplement: Supplementary file 2 — Additional file 2: Sensitivity and specificity for CDI-SF total scores in prediction of Kessler K6 cutoff for probable serious mental illness (K6 Australian scoring cutoff ≥18) [file 12872_2023_3439_MOESM2_ESM.docx]

**Additional file 2:** Sensitivity and specificity for CDI-SF total scores in prediction of Kessler K6 cutoff for probable serious mental illness (K6 Australian scoring cutoff ≥18)

| CDI-SF  Cutpoint | Sensitivity | Specificity | Correctly classified | LR+ | LR- |
| --- | --- | --- | --- | --- | --- |
| ( >= 0 ) | 100.00% | 0.00% | 15.01% | 1 |  |
| ( >= 1 ) | 100.00% | 13.47% | 26.46% | 1.1557 | 0.0000 |
| ( >= 2 ) | 100.00% | 20.96% | 32.82% | 1.2652 | 0.0000 |
| ( >= 3 ) | 100.00% | 29.04% | 39.69% | 1.4093 | 0.0000 |
| ( >= 4 ) | 100.00% | 35.03% | 44.78% | 1.5392 | 0.0000 |
| ( >= 5 ) | 98.31% | 42.81% | 51.15% | 1.7191 | 0.0396 |
| ( >= 6 ) | 98.31% | 48.80% | 56.23% | 1.9201 | 0.0347 |
| ( >= 7 ) | 96.61% | 53.59% | 60.05% | 2.0818 | 0.0633 |
| ( >= 8 ) | 96.61% | 58.98% | 64.63% | 2.3553 | 0.0575 |
| ( >= 9 ) | 96.61% | 61.98% | 67.18% | 2.5408 | 0.0547 |
| ( >= 10 ) | 96.61% | 67.07% | 71.50% | 2.9334 | 0.0505 |
| ( >= 11 ) | 96.61% | 71.56% | 75.32% | 3.3966 | 0.0474 |
| ( >= 12 ) | 93.22% | 75.15% | 77.86% | 3.7513 | 0.0902 |
| ( >= 13 ) | 84.75% | 80.54% | 81.17% | 4.3546 | 0.1894 |
| ( >= 14 ) | 83.05% | 82.63% | 82.70% | 4.7826 | 0.2051 |
| ( >= 15 ) | 79.66% | 85.93% | 84.99% | 5.6610 | 0.2367 |
| ( >= 16 ) | 76.27% | 88.02% | 86.26% | 6.3686 | 0.2696 |
| ( >= 17 ) | 74.58% | 89.52% | 87.28% | 7.1167 | 0.2840 |
| ( >= 18 ) | 66.10% | 92.51% | 88.55% | 8.8312 | 0.3664 |
| ( >= 19 ) | 64.41% | 93.71% | 89.31% | 10.2438 | 0.3798 |
| ( >= 20 ) | 55.93% | 94.01% | 88.30% | 9.3407 | 0.4687 |
| ( >= 21 ) | 49.15% | 94.91% | 88.04% | 9.6570 | 0.5357 |
| ( >= 22 ) | 42.37% | 97.01% | 88.80% | 14.1525 | 0.5941 |
| ( >= 23 ) | 38.98% | 97.60% | 88.80% | 16.2754 | 0.6251 |
| ( >= 24 ) | 37.29% | 98.20% | 89.06% | 20.757 | 0.6386 |
| ( >= 25 ) | 30.51% | 98.50% | 88.30% | 20.3796 | 0.7055 |
| ( >= 26 ) | 23.73% | 98.80% | 87.53% | 19.8135 | 0.7720 |
| ( >= 27 ) | 13.56% | 98.80% | 86.01% | 11.322 | 0.8749 |
| ( >= 28 ) | 8.47% | 99.40% | 85.75% | 14.1526 | 0.9208 |
| ( >= 30 ) | 6.78% | 99.40% | 85.50% | 11.3221 | 0.9378 |
| ( >= 31 ) | 6.78% | 100.00% | 86.01% |  | 0.9322 |
| ( >= 33 ) | 5.08% | 100.00% | 85.75% |  | 0.9492 |
| ( >= 35 ) | 3.39% | 100.00% | 85.50% |  | 0.9661 |
| ( >= 36 ) | 1.69% | 100.00% | 85.24% |  | 0.9831 |
| ( > 36 ) | 0.00% | 100.00% | 84.99% |  | 1 |

Observations = 393; ROC Area = 0.913, 95% CI (0.879-0.948); LR+ = Positive Likelihood Ratio=Sensitivity/(1-Specificity) ; LR- = Negative Likelihood Ratio=(1- Sensitivity)/Specificity
